# Clinical Value of Natriuretic Peptides in Predicting Time to Dialysis in Stage 4 and 5 Chronic Kidney Disease Patients

**DOI:** 10.1371/journal.pone.0159914

**Published:** 2016-08-22

**Authors:** Sofia Sundqvist, Thomas Larson, Bruno Cauliez, Fabrice Bauer, Audrey Dumont, Frank Le Roy, Mélanie Hanoy, Caroline Fréguin-Bouilland, Michel Godin, Dominique Guerrot

**Affiliations:** 1 Service de Néphrologie, CHU Hôpitaux de Rouen, Rouen, France; 2 Norwegian University of Science and Technology, Trondheim, Norway; 3 Service de Biochimie, CHU Hôpitaux de Rouen, Rouen, France; 4 Service de Cardiologie, CHU Hôpitaux de Rouen, Rouen, France; 5 INSERM Unité 1096, Université de Médecine-Pharmacie de Rouen, Rouen, France; Postgraduate Medical Institute, INDIA

## Abstract

**Background:**

Anticipating the time to renal replacement therapy (RRT) in chronic kidney disease (CKD) patients is an important but challenging issue. Natriuretic peptides are biomarkers of ventricular dysfunction related to poor outcome in CKD. We comparatively investigated the value of B-type natriuretic peptide (BNP) and N-terminal pro-B-type natriuretic peptide (NT-proBNP) as prognostic markers for the risk of RRT in stage 4 and 5 CKD patients, and in foretelling all-cause mortality and major cardiovascular events within a 5-year follow-up period.

**Methods:**

Baseline plasma BNP (Triage, Biosite) and NT-proBNP (Elecsys, Roche) were measured at inclusion. Forty-three patients were followed-up during 5 years. Kaplan-Meier analysis, with log-rank testing and hazard ratios (HR), were calculated to evaluate survival without RRT, cardiovascular events or mortality. The independent prognostic value of the biomarkers was estimated in separate Cox multivariate analysis, including estimated glomerular filtration rate (eGFR), creatininemia and comorbidities.

**Results:**

During the first 12-month follow-up period, 16 patients started RRT. NT-proBNP concentration was higher in patients who reached endpoint (3221 ng/L vs 777 ng/L, p = 0.02). NT-proBNP concentration > 1345 ng/L proved significant predictive value on survival analysis for cardiovascular events (p = 0.04) and dialysis within 60 months follow-up (p = 0.008). BNP concentration > 140 ng/L was an independent predictor of RRT after 12 months follow-up (p<0.005), and of significant predictive value for initiation of dialysis within 60 months follow-up.

**Conclusions:**

Our results indicate a prognostic value for BNP and NT-proBNP in predicting RRT in stage 4 and 5 CKD patients, regarding both short- and long-term periods. NT-proBNP also proved a value in predicting cardiovascular events. Natriuretic peptides could be useful predictive biomarkers for therapeutic guidance in CKD.

## Introduction

Predicting progression to end-stage renal disease (ESRD) in chronic kidney disease (CKD) patients is an important but challenging issue. To date, the clinical and biological tools helpful in foretelling the requirement for renal replacement therapy (RRT) are scarce and their reproducibility and predictive value are generally low.

CKD is associated with an elevated prevalence of cardiovascular (CV) disease, which is a major source of morbidity and mortality [1–3] The prevalence of left ventricular hypertrophy (LVH) and congestive heart failure (CHF) is exaggerated since the first stages of CKD [4, 5], and sharply increases with the decline of the glomerular filtration rate (GFR). Importantly, cardiovascular disease is associated with an accelerated evolution towards ESRD in CKD patients [6]. Natriuretic peptides are biomarkers of myocardial dysfunction. B-type natriuretic peptide (BNP) and amino-terminal pro-B-type natriuretic peptide (NT-proBNP) are the two major natriuretic peptides measured in routine analyses. Patients with CKD generally have raised levels of BNP and NT-proBNP, related both to volume overload and to the cardiovascular burden, including LVH and CHF [7–11]. In CKD, an elevated plasma concentration of these cardiac biomarkers is predictive of cardiovascular morbidity and all-cause death [8, 12].

In this prospective study, we comparatively evaluated the prognostic value of BNP and NT-proBNP in predicting short- and long-term RRT-requirement, as well as major CV events and all-cause mortality in stage 4 and 5 CKD patients.

## Methods

### Study population

This multicentric observational study recruited outpatients with CKD over a 22-month period of time, in Rouen and Dieppe nephrology departments, France. Patients with an estimated GFR (eGFR) < 30 mL/min/1.73m^2^ calculated by the 4-variable equation of the Modification of Diet in Renal Disease (MDRD) were included. Patients with acute coronary syndrome, acute heart failure, acute renal failure defined according to AKIN criteria, pre-established indication for RRT, unlikely RRT-requirement within 12 months, or conservative treatment decision, were excluded. A final cohort of 43 patients was followed-up during a 5-year period of time. The study was approved by the local ethics committee (CERNI E201435—CPP Nord Ouest 1).

### End points

The primary objective of the study was dialysis-free survival, stratified according to the median value of BNP at inclusion. The secondary objectives were the predictive value of NT-proBNP for RRT requirement, as well as the predictive value of both BNP and NT-proBNP for all-cause death, and a composite endpoint of cardiovascular events which included acute myocardial infarction, cerebral infarction or severe heart failure, defined as the need of intensive care unit admission, or acute initiation of dialysis.

### Procedures and measurements

The patients’ medical records were analyzed to obtain data on drugs, preexisting diabetes, hypertension, stroke, peripheral vascular disease or coronary artery disease (defined by history of myocardial infarction, angina pectoris or requirement for coronary artery bypass or angioplasty). Sex, age and body mass index were collected at inclusion and the 4-variable MDRD equation was used for calculation of the baseline estimated GFR (eGFR). Blood samples were collected at inclusion (M0). BNP, NT-proBNP, Troponin T (TnT) were detected in ethylenediaminetetraacetic acid plasma (EDTA) using a fluorescence immunoassay (Triage, Biosite) for measuring BNP and electro-chemiluminescence (Elecsys, Roche diagnostics) for measuring NT-proBNP and TnT. The blood samples were centrifuged and kept frozen at -80°C until 12 months after inclusion. Blood urea nitrogen, natremia, C reactive protein and hematocrit were measured with routine methods, within the four hours following the time of collection. Creatinine was measured from the same EDTA sample as the natriuretic peptides. The value of BNP and NT-proBNP were blinded to the patient’s nephrologist, in order to avoid potential bias in medical care. The time from enrollment to RRT, the indication and modalities of the first RRT were recorded. Information about hospitalization for acute coronary events, acute heart failure, stroke, or death within 5 years from enrollment was collected retrospectively.

### Statistical analyses

Statistical analyses were performed with Statview software version 5.0 (SAS Institute). A *p*-value<0.05 was considered significant. Comparisons were performed using the non-parametric Mann-Whitney *U* test. Receiver operator characteristic (ROC) curves for BNP and NT-proBNP were estimated to determine cut-off points at which sensibility and specificity was maximized. By stratifying the patients in two groups according to the plasma BNP and NT-proBNP levels above or below the cut-off values, survival curves were determined using the univariate Kaplan-Meier calculation and the significance between the curves tested using log-rank test. Kendall's tau coefficient was used to test for associations between BNP and other variables for inclusion in multivariate models.

## Results

### Patients’ characteristics

The characteristics of the 43 patients at inclusion are shown in Table 1. Median eGFR was 14.2 mL/min/1.73m^2^. Median concentrations of BNP and NT-proBNP were 134 ng/L, and 1127 ng/L, respectively.

**Table 1:** Patients’ characteristics at inclusion.

	n	Median Value *Or %*	Interquartile Range [Q1 –Q3]
Age (years)		76	63–78
Sex (male)	27	*63%*	
Coronary Artery Disease	12	*29%*	
Diabetes	20	*48%*	
Hypertension	41	*95%*	
Diuretics	32	*74%*	
BNP (ng/L)	43	134	38–367
NT-proBNP (ng/L)	42	1127	493–3935
TnT (ng/mL)	38	0.017	0–0.037
Plasma creatinine (μmol/L)	43	357	297–470
eGFR (mL/min/1.73m^2^	43	14.2	11.0–17.0
BUN (mmol/L)	43	29.2	22.2–37.8
Na^+^ (mmol/L)	43	139	137–140
Hct (%)	39	33.5	32.0–36.8
CRP ≥ 5 mg/L	20	*46*.*5%*	

### Prediction of time to dialysis

**Fig 1:**
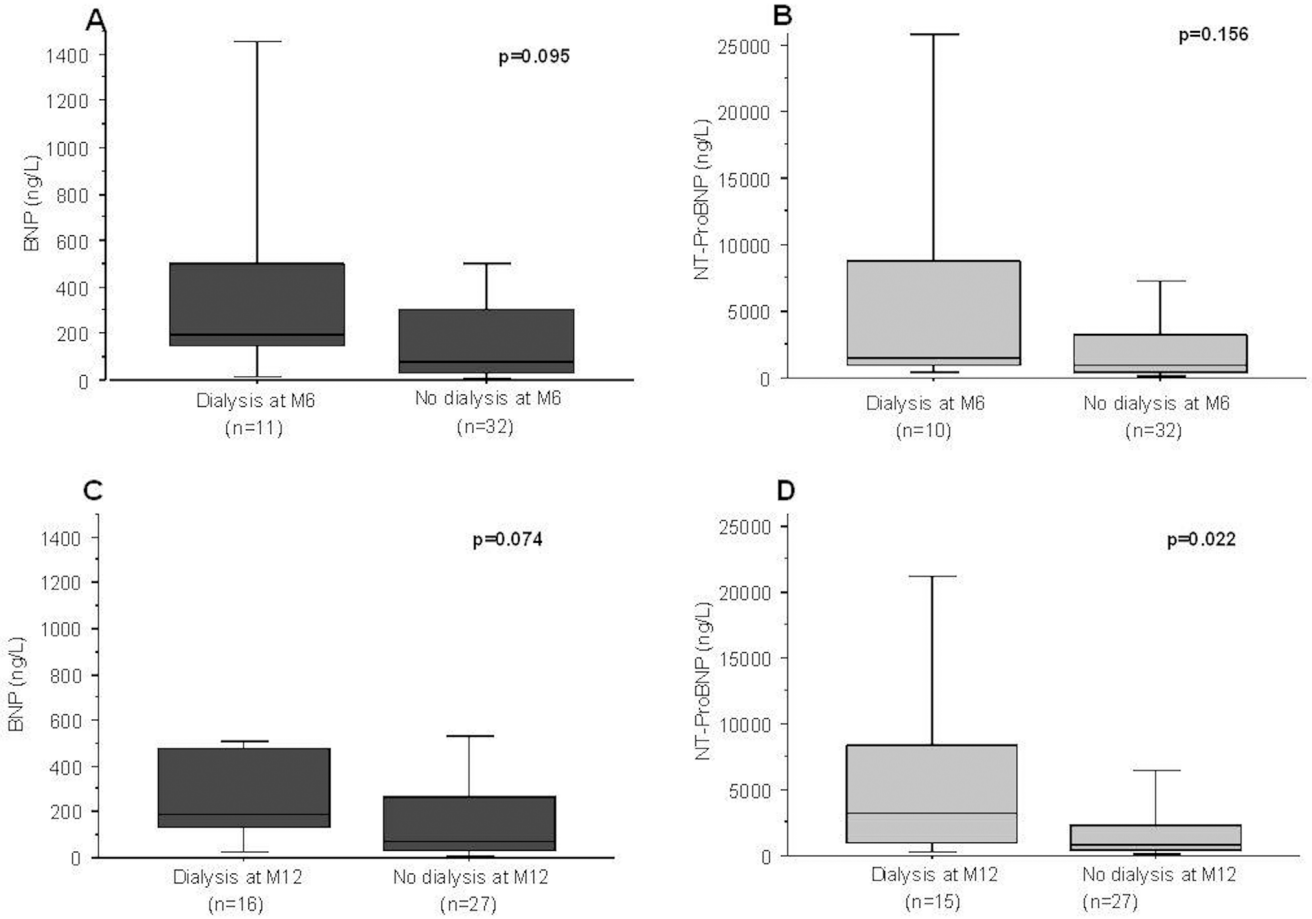
BNP (A, C) and NT-proBNP (B, D) values according to the initiation of dialysis after 6 (A, B), and 12 months (C, D) follow-up. The upper and lower limits represent the interquartile range (Q1-Q3) and the middle line of the box-plot represents the median. Differences between the groups were analyzed using the Mann-Whitney *U* test.

Dialysis was initiated in 16/43 patients after 12 months follow-up, and in 30/43 patients after 60 months. As shown in Fig 1, baseline plasma levels of both BNP and NT-proBNP were non-significantly higher in the patients who initiated dialysis within 6 months from inclusion (Fig 1A and 1B). The patients dialyzed after 12 months had a significantly higher baseline NT-proBNP concentration compared to dialysis-free patients (3221 ng/L (interquartile range 974–8381) vs 777 ng/L, p = 0.022) (Fig 1D). A similar result was found regarding BNP, but the difference was non significant (188 ng/L (133–479) vs 67 ng/L (40–265), p = 0.074) (Fig 1C). After 60 months follow-up, the latter differences between the 30 patients who had initiated dialysis and the dialysis-free patients were non-significant (p = 0.352 and p = 0.124 for BNP and NT-proBNP, respectively).

**Fig 2:**
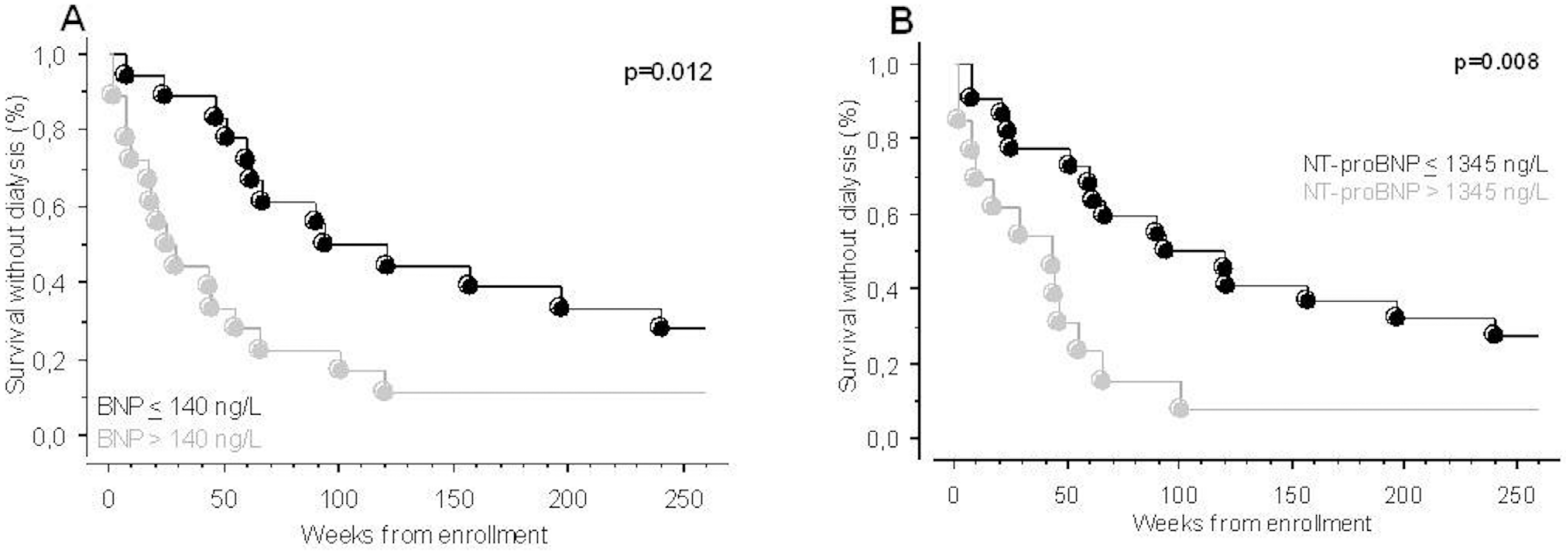
Kaplan Meier survival curves showing the association between survival and A) BNP above and below cut-off after 5 years follow-up. B) NT-proBNP above and below cut-off after 5 years follow-up. The difference between the groups was determined using the log-rank test.

**Fig 3:**
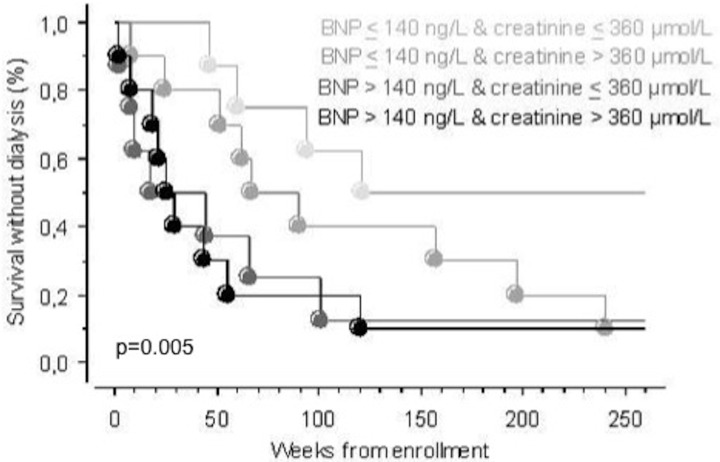
Kaplan Meier curves for survival without dialysis. The population is stratified according to BNP concentration above and below cut-off, as well as creatinine above and below the median, after 5 years follow-up. The significance indicated represents overall comparison.

Cut-off values were defined for BNP and NT-proBNP by ROC analysis as the crossing value of sensitivity and specificity curves for prediction of RRT-requirement after 12 months (140 ng/L and 1345 ng/L, for BNP and NT-proBNP, respectively). The patients were thereafter stratified into 2 groups according to these cut-off values to perform Kaplan Meier survival analyses. A BNP concentration >140 ng/L was a predictive factor for the initiation of dialysis within 12 months (p = 0.005) and 60 months (Fig 2A, p = 0.012). A NT-proBNP concentration >1345 ng/L was significantly predictive for the initiation of dialysis within 60 months (Fig 2B, p = 0.008), and marginally significant within 12 months (p = 0.053). A univariate Cox regression analysis was performed to calculate Hazard Ratios (HR) for RRT requirement within 12 months (Table 2). BNP was the only significant risk factor identified (HR = 4.5, p = 0.01). In this setting, NT-proBNP (HR = 2.7, p = 0.06), and plasma creatinine (HR = 1.9, p = 0.21) were not significantly associated with 12-month RRT requirement. In order to determine whether BNP may provide additional value to plasma creatinine, we stratified the study population according to BNP, and plasma creatinine median value (360 μmol/L). Patients with baseline creatinine >360 μmol/L and BNP >140 ng/L were at highest risk for having initiated dialysis after 12 months follow up (p = 0.005 for overall comparison) (Fig 3).

**Table 2:** Relative risk for initiation of dialysis within 12 months follow-up. Hazard ratios and 95% confidence intervals were calculated using univariate cox regression analysis.

	*Univariate Hazard Ratios* (CI 95%)	*p*
Age < 75 y	1.7 (0.6–4.5)	0.31
Sex (male)	1.9 (0.6–5.9)	0.27
Coronary artery disease	1.9 (0.7–5.2)	0.22
Diabetes mellitus	1.3 (0.5–3.4)	0.64
BNP > 140 ng/L	**4.5 (1.4–13.9)**	**0.01**
NT-proBNP > 1345 ng/L	2.7 (0.95–7.5)	0.06
TnT > 0.017 ng/mL	1.0 (0.3–3.1)	0.97
Plasma creatinine > 360 μmol/L	1.9 (0.7–5.3)	0.21
eGFR < 14.2 mL/min/1.73m^2^	1.2 (0.5–3.3)	0.69
BUN > 29 mmol/L	1.4 (0.5–3.8)	0.50
Na^+^ < 139 mmol/L	1.0 (0.3–2.8)	0.99
Hct ≤ 33%	0.7 (0.2–1.9)	0.43
CRP ≥ 5 mg/L	1.2 (0.5–3.3)	0.67

Kendall's tau coefficient revealed a significant association with BNP for continuous values of NT-proBNP (p<0.0001), age (p = 0.03), TnT (p = 0.02) and creatinine (p = 0.04). We therefore performed a mutivariate Cox proportional hazard model, including these variables, and age, sex, creatinine, and eGFR. BNP was an independent predictive factor for 12-month RRT requirement, with a HR of 5.9 (95%CI: 1.8–19.6; p<0.005).

**Fig 4:**
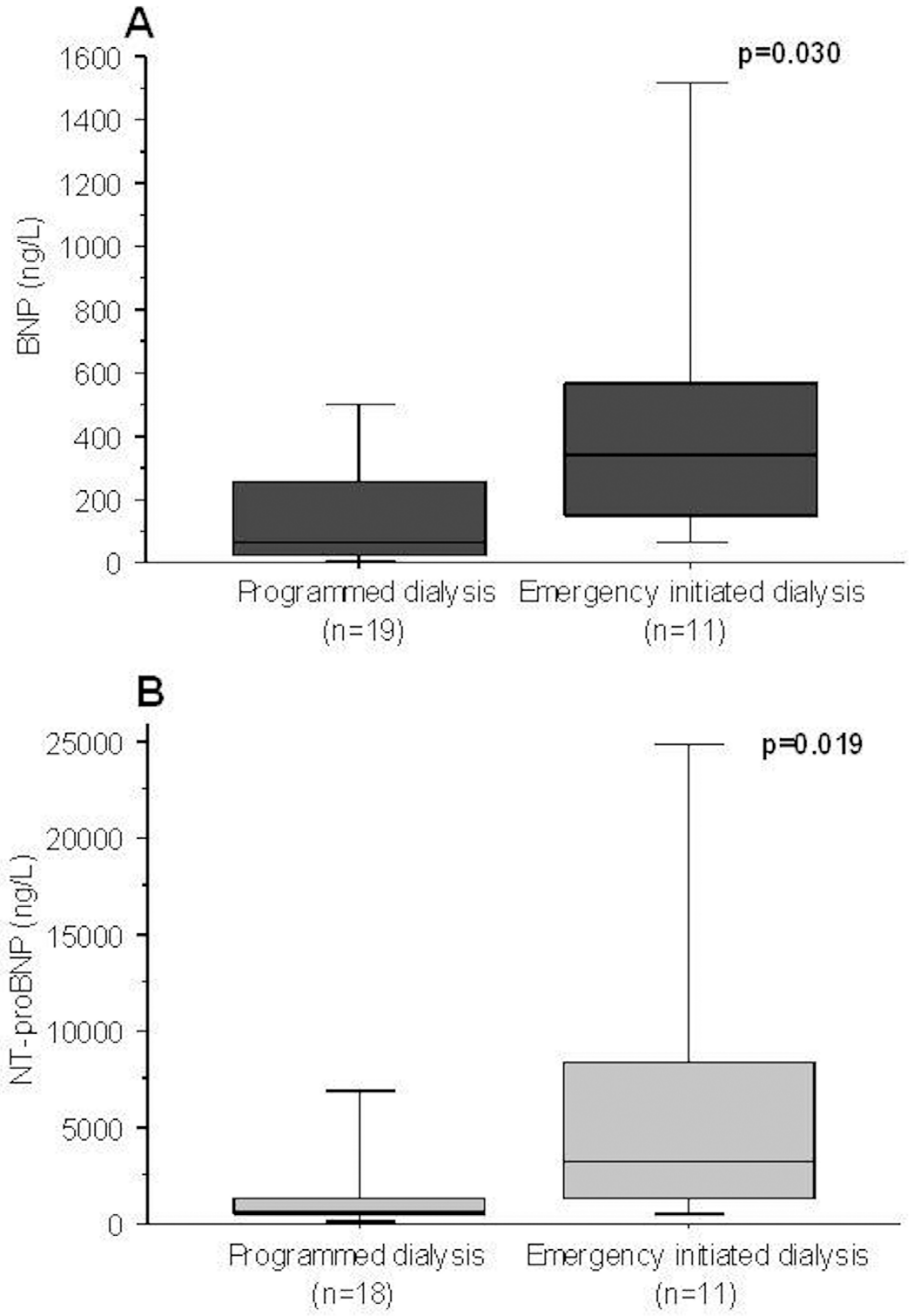
Baseline plasma natriuretic peptide concentrations for BNP (A) and NT-proBNP (B), according to the urgent or programmed dialysis initiation. Thirty patients had initiated dialysis within 5 years follow-up. Nineteen dialysis sessions were programmed and 11 were initiated urgently. Differences between the groups were analyzed using the Mann-Whitney *U* test.

At the end of follow-up, 2 patients had received a kidney transplant, and 14 patients had initiated RRT with hemodialysis performed on a native arterio-venous fistula, 7 with peritoneal dialysis on a peritoneal catheter, and 9 with hemodialysis on a central venous catheter (CVC), because of the absence of functional fistula or peritoneal catheter at RRT initiation. In 11 patients, dialysis was initiated in a situation of emergency. The main indications for emergency hemodialysis on a CVC were clinical symptoms related to uremia (n = 6), and pulmonary edema (n = 2). Interestingly, median baseline plasma natriuretic peptides in these 11 patients were significantly higher compared to those whose dialysis was planned (338 ng/L (147–588) vs 63 ng/L (28–277) p = 0.030 for BNP, and 3221 ng/L (1220–8762) vs 728 ng/L (410–2839) p = 0.019 for NT-proBNP) (Fig 4A and 4B).

### Prediction of all-cause mortality and cardiovascular events

During the follow-up period 9 patients died (21%), 2/9 due to major CV events. A Mann-Whitney *U* test did not identify baseline plasma concentrations of BNP or NT-proBNP to be significantly higher in those deceased, compared to the other patients (p = 0.387 and p = 0.154, respectively). Kaplan-Meier survival analysis did not reveal a significant association between BNP and all-cause mortality (p = 0.655), this association was not significant for NT-proBNP (p = 0.068).

**Fig 5:**
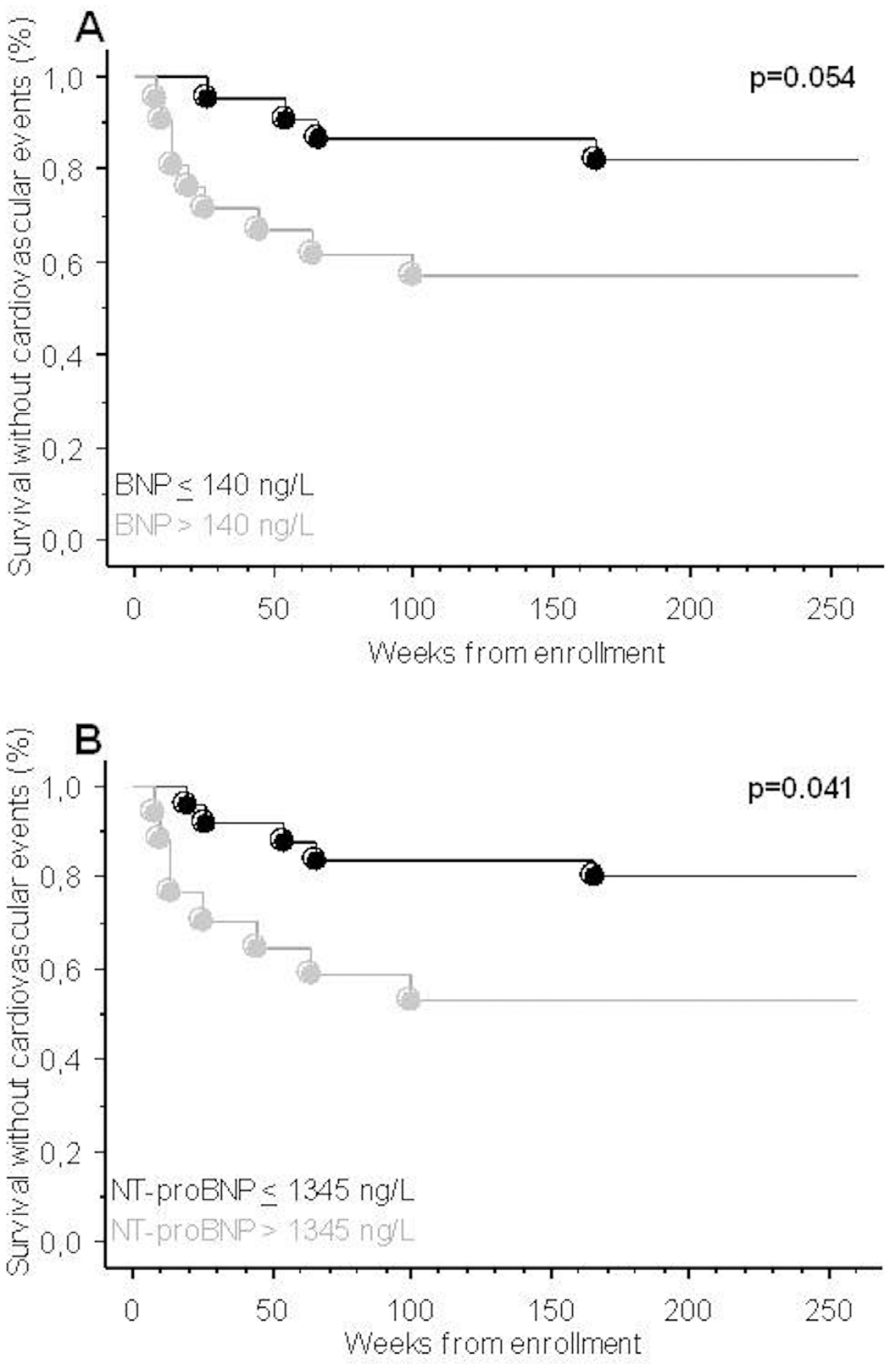
Kaplan-Meier survival analysis of BNP and NT-proBNP as associated factors with cardiovascular events. A) Curves for baseline BNP concentration above and below the cut-off value 140 ng/L. B) Curves for baseline NT-proBNP concentration above and below the cut-off value 1345 ng/L. The difference between the groups was determined using the log-rank test.

A total of 14 patients (33%) presented CV events after 60 months follow up (2 acute coronary syndromes, 2 strokes, and 10 pulmonary edemas). The survival analysis identified that baseline NT-proBNP > 1345 ng/L significantly predicted CV events (p = 0.041), while baseline BNP > 140 ng/L did not achieve statistical significance (p = 0.054) (Fig 5B and 5A).

## Discussion

This study shows that BNP and NT-proBNP are relevant biomarkers for short- and long-term prediction of dialysis requirement in stage 4 and 5 CKD patients.

Anticipating dialysis requirement during CKD progression is a major challenge for nephrologists. The optimal medical goal in this setting is to plan dialysis initiation on a functional arterio-venous fistula or peritoneal catheter, thereby avoiding emergency dialysis, and comorbidities related both to the medical condition and to CVC complications [13–16]. An arterio-venous fistula has to be created several weeks prior to hemodialysis, in order to be sufficiently mature. However, while data linking the presence of a fistula to heart failure, natriuretic peptides and eGFR decline are controversial, early creation of a fistula also carries risks, which should not be overlooked [13, 17–19]. Although not sufficient, a regular follow-up by a nephrologist is an important factor to determine the timing of the vascular or peritoneal access creation. This specialized follow-up has proven to be related to better outcomes in CKD patients [14, 15]. Currently, there are no objective criteria defining when the vascular or peritoneal dialysis should be created, and this decision is highly dependent on the physician’s experience, and the patient’s eGFR and symptoms. In this context, additional objective tools would be of highest interest.

In our study, 11 patients needed an emergency dialysis, 8 of which on a CVC, a condition related to important morbidity, mortality, and prolonged hospitalization [15, 16]. Our retrospective analysis showed that a BNP concentration >140 ng/L predicted the need of dialysis within 12 and 60 months. Our study did also show that both BNP and NT-proBNP were higher in patients that had to initiate emergency dialysis, compared to patients with planned dialysis. This suggests that the cardiovascular comorbidity of the CKD population included in this study was a major determinant of an unanticipated, rapidly progressive worsening of kidney function. The median concentrations in our study population were 134 ng/L for BNP and 1127 ng/L for NT-proBNP. This value of BNP is similar to the one reported by Takami *et al* (162 ng/L) in CKD patients with a comparable median eGFR (15.0 vs 14.2 mL/min/1.73m^2^) [20]. Tagore *et al* studied CKD stage 4 patients and they found median concentrations of 64 ng/L for BNP and 270 ng/L for NT-proBNP [21]. Vickery *et al* also showed median concentrations that were weaker than ours, with a median BNP of 48 ng/L and 576 ng/L for CKD stage 4 patients, and 73 ng/L for BNP and 1636 ng/L for NT-proBNP in stage 5 CKD [22]. When comparing the exlusion criteria between these studies and ours, our study population proved a higher prevalence of cardiovascular comorbidities. Furthermore, different measurement procedures have been used. This could possibly explain the differences between the values of natriuretic peptides in these studies.

When evaluating the utility of measuring BNP and NT-proBNP in foreseeing CV events and death, only NT-proBNP came out as statistically significant. A NT-proBNP above 1345 ng/L was a significant risk factor for initiating dialysis within 5 years follow-up, while a marginally significant trend was found in foretelling death. Other studies have proven both BNP and NT-proBNP to be predictive factors in this setting. Austin WJ *et al* have done a prospective study in 171 CKD patients, in which 54 were classified as stage 4 or 5 [23]. Within a mean follow up of 268 days, 15 patients experienced a CV event and 7 died. They found plasma concentrations above 175 pg/ml for BNP and 1250 pg/mL for NT-proBNP to be significantly predictive of a composite end-point including death and hospitalization for cardiac events. This cut-off was calculated with the goal to predict mortality and primary events for patients across different CKD stages, while our study was limited to stage 4 and 5 CKD patients. Furthermore, 37% of our patient enrolled were women, compared to 2% in their study. Horii M *et al* followed a group of 1083 patients with CV disease for a mean duration of 52 months [24]. Stratified by eGFR, 85 of these patients were in CKD stage 4 and 5 with a median eGFR of 12.4 mL/min/1.73m^2^. Their median BNP and NT-proBNP concentration was 178 pg/mL and 6220 pg/mL, respectively. They found optimal cut-off points for mortality to be 115 pg/mL for BNP, and 5809 pg/mL for NT-proBNP. Cut-offs for composite endpoint of cardiovascular disease were 157 pg/mL and 5111 pg/mL, respectively. When excluding patients on hemodialysis, cut-off values were lowered to 115 pg/mL for BNP and 4740 pg/mL for NT-proBNP, indicating a need for higher cut-offs for patients having already initiated dialysis. Interestingly, they found much lower cut-off values for the patients with an eGFR >30mL/min/1.73m2. When comparing to the cut-offs for CKD stages 4 and 5, the difference was up to 20 times higher for NT-proBNP, compared to only 30% higher for BNP. Horii M *et al* concluded that NT-proBNP could have a superior prognostic value than BNP in stage 4 and 5 CKD patients, based on comparative ROC analysis [24]. They performed BNP measurements with a different assay kit than ours (Shionogi *vs* Biosite), and the specificity for cut-offs can differ between different kits.

Interestingly, in our study, BNP held a higher predictive value compared to plasma creatinine in foretelling the need for dialysis initiation within both 12 months and 60 months follow-up periods. While plasma creatinine and its evolution are currently the main variables used to estimate the time to ESRD, this study suggests that natriuretic peptides could be of greater value to provide guidance for the anticipation of dialysis. Natriuretic peptides are influenced by multiple factors. BNP and NT-proBNP both originate from the same precursor, and are secreted from the left ventricular wall as a response to wall stress [25]. Accordingly, high plasma levels are seen in chronic heart failure and ventricular hypertrophy [26]. In a majority of CKD patients, plasma concentrations of BNP and NT-proBNP are increased as a combined consequence of diminished renal clearance, fluid overload, and high prevalence of comorbidities such as LVH, and systolic and diastolic heart dysfunction [27]. When both peptides are measured, NT-proBNP shows higher concentrations than BNP, even though both peptides are initially released in an equimolar fashion. BNP has approximately a 6-fold shorter half-life than NT-proBNP [27–29]. NT-proBNP clearance is mainly renal, while BNP is also cleared by its receptor, independently of GFR. NT-proBNP level is therefore considered to be more directly influenced by kidney function. [27]. Though both BNP and NT-proBNP increase progressively along with the diminishing GFR, the increase is proportionally higher for NT-proBNP [23]. Furthermore, sex and age also have an influence of the peptides’ plasma levels, with higher levels seen in females and in older patients [27]. Hence, these factors, which influence BNP and NT-proBNP concentrations, should be taken into account in clinical practice for the interpretation of individual patients values.

This prospective study has several limitations, notably the limited number of selected CKD patients, with a small number of cardiovascular endpoints reached over the 5-year study period. This may have underestimated the predictive potential of the biomarkers analyzed, especially when considering cardiovascular events and mortality. Blood measurements of BNP and NT-proBNP have been performed with Biosite and Roche respectively, and extrapolation of the results of this study must take inter-essay variability into account.

In conclusion, this study shows that BNP and NT-proBNP are of significant value in predicting the need for dialysis in CKD stage 4 and 5 patients. In this setting, our results suggest that natriuretic peptides may have a better predictive value compared to plasma creatinine. Natriuretic peptides could therefore be useful for therapeutic guidance in stage 4 and 5 CKD patients.
